# Feasibility of Anesthesiologists Giving Nurse Anesthetists 30-Minute Lunch Breaks and 15-Minute Morning Breaks at a University’s Facilities

**DOI:** 10.7759/cureus.25280

**Published:** 2022-05-24

**Authors:** Sarah S Titler, Franklin Dexter

**Affiliations:** 1 Department of Anesthesia, University of Iowa, Iowa City, USA

**Keywords:** staff assignment, surgical times, operating room management, industrial engineering, anesthesiology

## Abstract

Background

Managers of an anesthesia department sought an estimation of how often each anesthesiologist can give lunch breaks and morning breaks to nurse anesthetists to plan staff scheduling. When an anesthesiologist supervising the nurse anesthetists can give a break, it would be preferred because fewer extra nurse anesthetists would be scheduled to facilitate breaks.

Methodology

Our methodological development used retrospective cohort data from the three surgical suites of a single anesthesia department. Surgical times were estimated using three years of data from October 2016 through September 2019, with 95,146 cases. Comparison was made with the next year from October 2019 through September 2020, with 30,987 cases. The 5% lower prediction bounds for surgical time were estimated based on two-parameter, log-normal distributions. The times when two and three sequential rooms had overlapping lower prediction limits were calculated. Sequential rooms were used because that was how anesthesiologists’ assignments were made at the studied department, when feasible given constraints. Percentages of cases were reported with 15 minutes available starting sometime between 9:00 and 10:30 and 30 minutes starting sometime between 11:15 and 12:45, times characteristic for the studied department. At the studied university’s facilities, the nurse anesthetists were independent practitioners (e.g., an anesthesiologist supervising two nurse anesthetists each with a long case could give a break to one of the two rooms).

Results

The percentage of days for which an anesthesiologist could give a lunch break (11:15-12:45) was close to the percentage of cases when an anesthesiologist could give the same-length break anytime throughout the workday. In other words, the length of the break was important, not the time of the day of the break. The absolute percentages also depended on how many rooms the anesthesiologist supervised, the duration of cases, and facility. For example, among anesthesiologists at the adult surgical suite supervising three nurse anesthetists, a lunch break could be given by the anesthesiologist on at most one-third of the days without affecting workflow.

Conclusions

Our results show that the feasibility of an anesthesiologist clinically supervising one, two, or three rooms to give lunch breaks to the nurse anesthetists in the rooms depends principally on how many rooms are supervised, the duration of the break, and the facility’s percentage of cases with surgical times longer than that duration. The specific numerical results will differ among departments. Our methodology would be useful to other departments where anesthesiologists are clinically supervising independent practitioners, sometimes during cases long enough for a break, and there is anesthesiologist backup help. Such departments can use our methodology to plan their staff scheduling for additional nurse anesthetists to give the remaining breaks.

## Introduction

Staff scheduling of nurse anesthetists for large group practices includes planning some practitioners to give breaks. To help that planning, an anesthesia department with anesthesiologists and nurse anesthetists collaboratively caring for patients aimed to estimate accurately how often each anesthesiologist can give lunch and morning breaks to nurse anesthetists with both present at anesthesia induction and emergence. For example, at the studied department’s pediatric hospital, often an anesthesiologist would clinically supervise two nurse anesthetists each caring for pediatric patients during long-duration surgery. Sometimes an anesthesiologist can give a lunch break to one of the two nurse anesthetists supervised. We evaluated how to perform these calculations using the probability distributions of surgical times [1], limiting consideration to specific time periods in the workday. The results provided the department with information on how many additional nurse anesthetists to schedule daily for the remaining breaks. Our results and methodology will be useful for other departments with the same personnel structure and governing laws or regulations on supervision.

## Materials and methods

Our university’s Institutional Review Board determined that this project [1,2] does not meet the regulatory definition of human subjects research because the activity was limited to the analysis of de-identified data provided for routine administrative purposes. Three facilities of one large university group practice were studied (Table 1).

**Table 1:** Anesthesiologists giving 30-minute breaks during the 1.5-hour lunchtime period. ^a^The sample sizes refer to the numbers of calculated 5% lower prediction limits of surgical procedure times. This was slightly less than the number of cases [1]. ^b^Column entries are percentage (%) chances of being able to give one break among the rooms, between 15 minutes after the start of surgical procedure(s) and the end of the surgical procedure(s), along with 99% lower and upper confidence intervals [1]. ^c^The “best chance” means the case is paired with the adjacent room giving the largest amount of overlapping time. The “least chance” means the other room. These columns with two rooms can be applied in the United States as breaks for trainees. All columns are predicated on there being another anesthesiologist available as a backup if the anesthesiologist is alone, giving a lunch break. ^d^The timing “throughout the workday” refers to that appropriate for a breast milk pumping session [1] because women a few months after giving birth would often need three sessions during a full (>10-hour) workday. These results were recalculated and correctly match those reported previously for breast milk pumping sessions [1], matching because both breaks are planned for 30 minutes. ^e^These two values being the same is not a mistake, it just happened to be so.

Location	Sample size^a^	Single cases^b^	Two rooms, best chance^b,c^	Two rooms, least chance^b,c^	Three rooms^b^
Ambulatory surgery center, adult, throughout the workday^d^	7,987	18.0 (16.9, 19.1)	16.6 (15.5, 17.6)	10.5 (9.6, 11.4)	10.3 (9.4, 11.2)
Ambulatory surgery center, break starting 11:15 to 12:45	1,459	17.3 (14.9, 20.0)	16.4 (14.0, 19.1)	10.9^e^ (8.9, 13.2)	10.9^e^ (8.9, 13.2)
Children’s hospital, throughout the workday^d^	4,872	20.7 (19.2, 22.2)	19.1 (17.6, 20.6)	13.1 (11.9, 14.4)	12.7 (11.5, 14.0)
Children’s hospital, break starting 11:15 to 12:45	670	18.5 (14.8, 22.7)	17.8 (14.1, 21.9)	12.2 (9.2, 15.8)	11.6 (8.7, 15.2)
Adult inpatient surgical suite, throughout the workday^d^	17,498	54.2 (53.2, 55.2)	51.5 (50.6, 52.5)	36.1 (35.2, 37.1)	31.4 (30.5, 32.3)
Adult surgical suite, break starting 11:15 to 12:45	1,979	50.9 (48.0, 53.8)	50.1 (47.2, 53.0)	36.6 (33.8, 39.4)	34.7 (32.0, 37.5)

The historical period to estimate future surgical times was three years from October 2016 through September 2019 [1,2]. We used every one of the 95,146 cases performed at the university’s surgical suites, with no exclusions, to predict future surgical times [1,2]. A comparison was done with the cases performed in the next (future) year from October 2019 through September 2020, limited to the studied surgical suites (Table 1) [1,2]. Surgical cases of the same procedure were matched between the historical three-year period and the future one-year period. The primary surgical Healthcare Common Procedure Coding System code was used. This procedure coding system is the one used for federal payment of hospitals in the United States. Surgical time was the time from the documented procedure start time, most typically incision, to the procedure end time, most typically applying dressings on the patient [3].

Prediction bounds

A prediction bound for a single future observation is a value that will, with a specified degree of confidence, be exceeded by the next randomly selected observation from a population. A 5% lower prediction bound for a given case gives a 5% risk of the surgical time being too brief for the break. For each procedure, the historical data provided a sample size, sample mean of logarithms of surgical times, and sample standard deviation of the logarithms of surgical times [1,2,4]. For procedures with more than 99 previous cases, the most recent 99 cases were used [1-3]. The 5% lower prediction bound was calculated in the log scale using the Student’s t-distribution [1-3]. Then, the exponential was taken for the result to convert the value into units of hours [1-3]. The observed incidence achieved by our 5% lower prediction bounds was 4.86% (99% confidence interval: 4.55% to 5.19%) [1,2]. All confidence intervals were 99% conservative Clopper-Pearson two-sided intervals for percentages [1].

The start of the available time for the break was considered to be 15 minutes after the start of surgery [1,2,5]. Ideally, anesthesia induction would be complete, patient positioning would be complete, and initial documentation would be finished. These steps were complete in 50% of cases 12 to 13 minutes after surgical incision, depending on the surgical procedure, insensitive to the surgical time, type of anesthetic, positioning, and facility [4]. Those results were why we used the longer period of 15 minutes. The end time for the break was considered the time of the start of surgery plus the 5% lower prediction bound minus the 15 minutes at the start of surgery, unless the case finished sooner, in which event it was when the case finished [1,2]. (Note that our calculations are meant to be useful for other departments where both anesthesiologists and nurse anesthetists are expected to be present at induction and emergence. In departments where that condition does not apply, our methodology would not apply.)

The facilities’ sequentially numbered operating rooms were treated as having cyclic adjacency [1]. For each case’s two adjacent rooms, the maximum overlapping period was calculated [1]. Because there were two options for two rooms, the minimum (“least chance”) and maximum (“best chance”) were reported [1]. When there was no case in the adjacent room during the period suitable for the break, it was treated as the best chance because the anesthesiologist was considered as having only one ongoing case [1]. For three rooms, both adjacent rooms were used [1]. (Note that at the studied department, when feasible given constraints, anesthesiologists are assigned to supervise rooms that are adjacent to one another. In other departments where that is not so and walking time would need to be added when considering the feasibility of giving breaks, then our methodology would not apply unless walking time were added.)

Hypothesis

The studied times in the workday were 11:15 to 12:45 for the lunch break (Table 1) and 9:00 to 10:30 for the mid-morning break (Table 2). Our hypothesis was based on the conceptually similar problem of having staff available to give breast milk pumping session breaks to anesthesia practitioners who are lactating [1,2]. For example, suppose that an anesthesiologist has just returned from maternity leave and will therefore need relief for breast milk pumping sessions. How often can a second anesthesiologist give relief for a breast milk pumping session during periods of the first anesthesiologist’s simultaneous cases such that she is present during the critical periods at the start and end of each case and has time to report on each case [1]? A period of 30 minutes was previously considered for a breast milk pumping session [1,2]. What we recognized is that the interval can be considered to match the time for the first anesthesiologist instead to give a lunch break to a nurse anesthetist, with the second anesthesiologist being available for emergency support. Therefore, our hypothesis was that results for lunch breaks would be similar to results for breast milk pumping sessions, especially that there would be large heterogeneity of calculated results [1,2] among facilities.

**Table 2:** Anesthesiologists giving 15-minute breaks mid-morning over a 1.5-hour period. ^a^The sample sizes refer to the numbers of calculated 5% lower prediction limits of surgical procedure times. This was slightly less than the number of cases [1]. ^b^Column entries are percentage (%) chances of being able to give one break among the rooms, between 15 minutes after the start of surgical procedure(s) and the end of the surgical procedure(s), along with 99% lower and upper confidence intervals [1]. ^c^The “best chance” means the case is paired with the adjacent room giving the largest amount of overlapping time. The “least chance” means the other room. These columns with two rooms can be applied in the United States as breaks for trainees. All columns are predicated on there being another anesthesiologist available as a backup if the anesthesiologist is alone, giving a morning break. ^d^The timing “throughout the workday” could refer to that appropriate for a breast milk pumping session [1] because women a few months after giving birth would often need three sessions during a full (>10-hour) workday. However, the 15-minute duration of the current table is insufficient for breast milk pumping. ^e^Breaks could be more often given in the morning than if distributed randomly throughout the workday. This result was caused by the period 9:00 to 10:30 being too early in the workday for many of the long surgical procedures performed at the adult inpatient surgical suite to be ending.

Location	Sample size^a^	Single cases^b^	Two rooms, best chance^b,c^	Two rooms, least chance^b,c^	Three rooms^b^
Ambulatory surgery center, adult, throughout the workday^d^	7,987	33.3 (31.9, 34.7)	32.0 (30.7, 33.4)	24.1 (22.9, 25.4)	23.5 (22.3, 24.7)
Ambulatory surgery center, break starting 9:00 to 10:30	1,492	30.8 (27.7, 33.9)	30.3 (27.3, 33.5)	23.1 (20.3, 26.0)	22.2 (19.5, 25.1)
Children’s hospital, throughout the workday^d^	4,872	33.0 (31.2, 34.7)	31.8 (30.0, 33.5)	25.5 (23.9, 27.1)	24.6 (23.0, 26.2)
Children’s hospital, break starting 9:00 to 10:30	985	40.5 (36.5, 44.6)	38.8 (34.8, 42.9)	29.2 (25.6, 33.1)	27.3 (23.7, 31.1)
Adult inpatient surgical suite, throughout the workday^d^	17,498	65.9 (64.9, 66.8)	64.7^e^ (63.8, 65.7)	53.6^e^ (52.6, 54.5)	45.3 (44.3, 46.3)
Adult surgical suite, break starting 9:00 to 10:30	3,854	78.7 (76.9, 80.3)	80.2^e^ (78.5, 81.8)	66.4^e^ (64.4, 68.4)	45.3 (43.2, 47.4)

We were (are) unaware, however, of any basis for presupposing that results for breast milk pumping sessions made across the entire day [1,2] would apply to specific 1.5-hour intervals (Tables 1, 2). On the contrary, operating rooms in use and durations of turnovers vary markedly among times of the workday [6-8]. Therefore, to allow comparison of results, the same (historical) data were used for current calculations. Demographics (e.g., surgical specialties) are presented by Titler et al. [2]. The results throughout the day are included in Table 1 and Table 2. (Note that the results for a break anytime during the day in Table 2 have no relevance to breast milk pumping sessions because 15 minutes is too brief. Nonetheless, the results are included in Table 2 for comparison of results, addressing whether results are sensitive to the time of the day of the break.)

Sensitivity analyses

Both the mid-morning and lunch breaks were represented as starting during a 1.5-hour period to allow comparison of results between 30-minute versus 15-minute breaks during the same available time. We extended the period of breaks from 1.5 hours to 2.0 hours (Table 3).

**Table 3:** Specific numerical results depend on the duration of periods available for 30-minute breaks or breast milk pumping sessions. ^a^The sample sizes refer to the numbers of calculated 5% lower prediction limits of surgical procedure times. This was slightly less than the number of cases [1]. ^b^Column entries are percentage (%) chances of being able to give one break among the rooms, between 15 minutes after the start of the surgical procedure(s) and the end of the surgical procedure(s), along with 99% lower and upper confidence intervals [1]. ^c^The “best chance” means the case is paired with the adjacent room giving the largest amount of overlapping time. The “least chance” means the other room. These columns with two rooms can be applied in the United States as breaks for trainees. All columns are predicated on there being another anesthesiologist available as a backup if the anesthesiologist is alone, giving a lunch break. ^d^These rows are from Table 1.

Location	Sample size^a^	Single cases^b^	Two rooms, best chance^b,c^	Two rooms, least chance^b,c^	Three rooms^b^
Ambulatory surgery center, break starting 11:15 to 12:45^d^	1,459	17.3 (14.9, 20.0)	16.4 (14.0, 19.1)	10.9 (8.9, 13.2)	10.9 (8.9, 13.2)
Ambulatory surgery center, break starting 11:00 to 13:00	1,929	18.2 (16.0, 20.6)	17.2 (15.1, 19.5)	11.6 (9.8, 13.6)	11.5 (9.7, 13.5)
Children’s hospital, break starting 11:15 to 12:45^d^	670	18.5 (14.8, 22.7)	17.8 (14.1, 21.9)	12.2 (9.2, 15.8)	11.6 (8.7, 15.2)
Children’s hospital, break starting 11:00 to 13:00	920	19.2 (16.0, 22.8)	17.8 (14.7, 21.3)	12.5 (9.8, 15.6)	12.1 (9.4, 15.1)
Adult surgical suite, break starting 11:15 to 12:45^d^	1,979	50.9 (48.0, 53.8)	50.1 (47.2, 53.0)	36.6 (33.8, 39.4)	34.7 (32.0, 37.5)
Adult surgical suite, break starting 11:00 to 13:00	2,587	51.3 (48.8, 53.9)	50.5 (48.0, 53.1)	36.3 (33.8, 38.7)	34.4 (32.0, 36.9)

We evaluated the sensitivity of results to the staff scheduling of the nurse anesthetists using the middle-sized (Children’s Hospital) surgical suite. Predictions would be made for single rooms by a combination of service and day of the week for use in staff scheduling of the few (individual) nurse anesthetists with special needs for relief (e.g., nurse anesthetist needs breast milk pumping sessions). Among the Children’s Hospitals’ 4,872 cases (Table 1), there were 50 combinations of service (e.g., orthopedics) and weekdays (Mondays to Fridays) with at least five cases. Being statistically independent comparisons, Šidák correction was used to achieve a family-wise error rate of 5%. We calculated the resulting 99.9% lower one-sided Clopper-Pearson confidence limits for the percentages of cases starting at any time of the day and with sufficient time for a 30-minute break.

## Results

**Figure 1:**
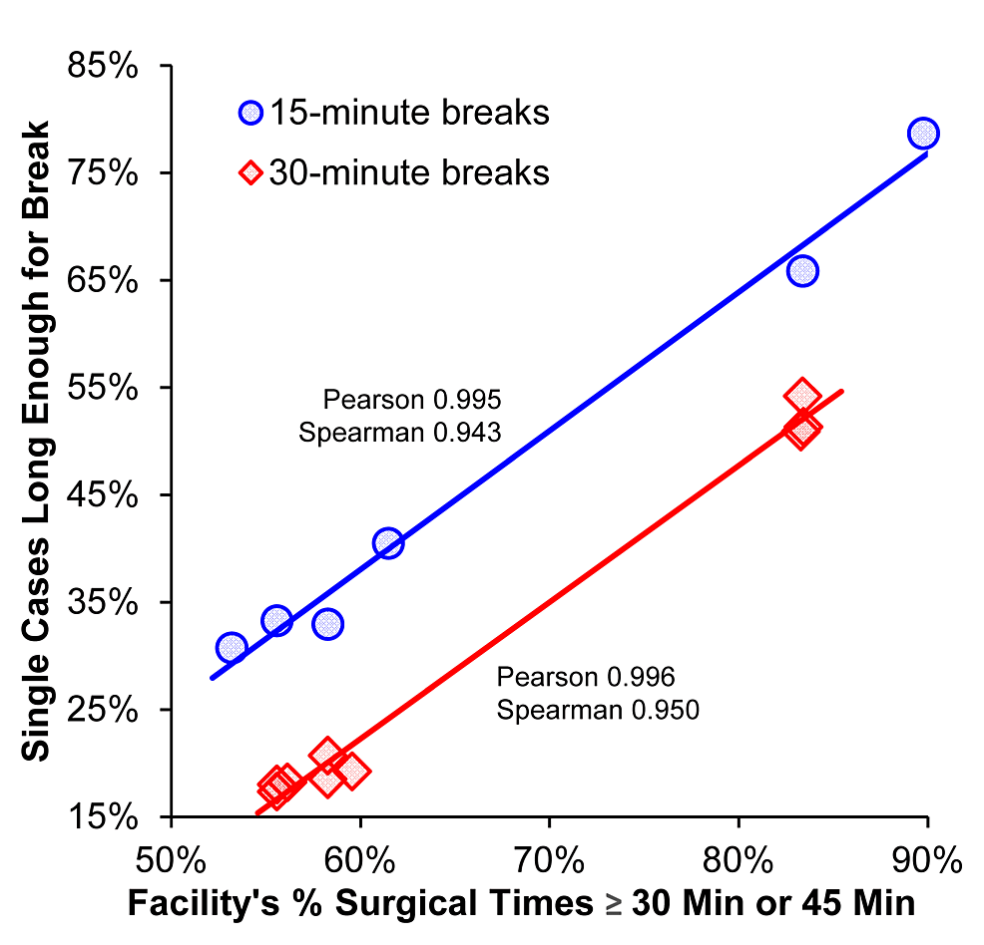
Association between characteristics of the facility and results by the facility. The factors important for the percentages of days for which an anesthesiologist could give lunch break were the number of cases being supervised, duration of the break, and facility. The figure explores the generalizability of the results. Along the vertical axis are plotted values for single cases in Table 2 (blue dots), Table 1 (red dots), and Table 3 (red dots). Along the horizontal axis are the facility’s percentages of surgical cases during the selected time of day that were at least 30 minutes (for 15-minute breaks) and 45 minutes (for 30-minute breaks). The data deliberately were not jittered. The data are nearly monotonic, as shown by the Spearman rank correlation coefficients being 0.950 and 0.943, respectively. Yet, linear correlation matters from the perspective of understanding the facility as a covariate. The Pearson correlation coefficients were 0.996 and 0.995, respectively. The implication is that, to the extent discernable from the N = 15 combinations of the facility and the time of day, the characteristic of facility affecting results is the percentage of cases with surgical time at least as long as that of the break, plus the 15 minutes used at the start for patient stabilization, positioning, finishing documentation, etc. [4]. The figure also highlights that the percentage of cases when an anesthesiologist supervising just one room can give a lunch break (and have both present waking up the patient) is less than the percentage of cases at the facility with surgical times at least of the length of the break. For example, from the last row of Table 1 and the end of the red line in the figure, for a nurse anesthetist to have a near 50:50 chance of receiving a 30-minute lunch break between 11:15 and 12:45, the facility needs to have overall 83% or more cases with surgical times of 45 minutes or longer. The reason the percentage probabilities along the vertical axis are smaller is that the true duration of surgical time is known only in retrospect.

The percentages of days for which an anesthesiologist can give a lunch break depends, of course, on how many rooms the anesthesiologist supervises (i.e., columns of the tables) and the duration of cases (i.e., rows of the tables). For example, from the lower right-hand corner of Table 1, among anesthesiologists at the adult surgical suite supervising three nurse anesthetists, a lunch break could be given at most one-third of the days. The percentages are larger for briefer 15-minute breaks (Table 2). Comparing sequential rows of Table 1, the percentages of days for giving a lunch break were close to percentages of cases with the same 30-minute duration but without regard to the time of the workday. Comparing sequential rows of Table 2, that finding also applied to morning breaks. Table 3 shows that consideration of cases suitable for a break during a longer 2.0-hour period versus the preceding 1.5-hour periods resulted in percentages that were only slightly different, with the observed absolute differences of <1%. Thus, key factors were the number of rooms being supervised, duration of the break, and facility, but not the time of the day or the period suitable for the break (Figure 1).

Revising staff scheduling of nurse anesthetists might be of value to facilitate breaks of a few individuals when done among facilities (i.e., more opportunities in the adult surgical suite versus the ambulatory surgery center) (Tables 1-3) [9], but not within facilities. For the Children’s Hospital’s 50 service and weekday combinations, only pediatric cardiac surgery on three weekdays had at least half the cases long enough for a nurse anesthetist reliably to have a 30‑minute break. However, only a few nurse anesthetists were assigned those cases given their specialization.

## Discussion

Specific results will differ among facilities, just like they varied among the studied department’s surgical suites, because of differences in surgical times [3]. For example, facilities that are not teaching programs will have smaller percentages of cases suitable for breaks. Our report shows the methodology that other departments can use to calculate the maximum percentage feasibility of anesthesiologists giving 30-minute lunch breaks and 15-minute morning breaks to nurse anesthetists while being present during critical portions of the case [1,2]. The percentages are maximum because sometimes there are (obviously) major intraoperative events during surgical periods. The results for lunch breaks may be especially useful because that period is the second busiest time of the day for supervising anesthesiologists after the first case of the day starts [10].

Our tables show the (obvious) dependence on durations of cases at different facilities, less opportunity for breaks at the ambulatory surgery center with brief cases, and more opportunity at the inpatient surgical suite with long cases. We expect generalizability based on the characteristics of the individual procedures at each facility because the principal factor influencing lower prediction limits relative to average durations is the sample size of historical data by the procedure [11,12]. The 5% lower prediction bounds were calculated by procedure, thus mitigating the effect of individual surgeons having small historical sample sizes for some procedures [13]. Uncommon procedures at one facility (e.g., one Children’s Hospital) tend to be uncommon at other such facilities [14,15], and there are uncommon procedures observed at many facilities [16,17]. In contrast, facilities have vastly different numbers of breaks and handoffs, reflecting personnel, culture, and organizational aims [18,19]. There would also be a vastly reduced need for breaks during cases if there were scheduled gaps between cases exceeding routine turnover times (e.g., first surgeon in the room estimated to finish their list one or two hours before when the second surgeon is scheduled to arrive from the morning clinic) [20,21]. The calculations can be useful for organizations with anesthesiologists’ supervision referring to clinical care not billing (i.e., when anesthesiologists bill for time, instead follow contract rules). The results presented in tables for pairs of rooms can apply to breaks for trainees in countries such as the United States where permitted.

Limitations

Given the results were from three surgical suites from one department, we cannot reliably know whether the characteristics of facilities affecting results can be limited solely to the observed percentages of surgical cases comparably long. However, Figure 1 shows that may very well be so. Simply put, the percentages of surgical cases suitable for breaks are considerably less than that known in retrospect because the reality is that a case must be estimated to be considerably longer to have a 95% probability of lasting long enough for both the anesthesiologist and nurse anesthetist to be present at emergence [2].

Our results should not be interpreted as suggesting value in anesthesiologists giving (or not giving) breaks because much of anesthesiologists’ activity is for subsequent and completed anesthetics [22-26]. For example, approximately half of the pages to anesthesiologists supervising operating rooms are from outside the rooms (e.g., preoperative holding area) [21]. Most emergency pages are from outside the operating rooms (e.g., bradyarrhythmia during intravenous line placement) [22]. Most episodes of hypoxemia in the phase I post-anesthesia care unit occur after the anesthesia provider has left the patient [23]. Operating room control desks at hospitals average more than or equal to one inpatient cancelation or add-on case scheduled per hour during regular workdays [24]. Moreover, when preoperative evaluations are completed on the day of surgery, turnover times are increased unless completed before the earlier case in the room is complete [25]. Our methodology would be useful for other departments where the decision has been made that when feasible an anesthesiologist clinically supervising rooms will give some breaks. The department administration then seeks guidance on how many additional nurse anesthetists should be scheduled for additional (and from our results generally the majority) breaks. Inherently, because usefulness depends on the anesthesiologist giving some breaks when feasible, our results and methodology depend on the department meeting the governing laws and regulations, including billing.

Our results should not be interpreted as suggesting that cases need to have both anesthesiologists and nurse anesthetists at the end of the case. Similarly, what we treated as best was for the anesthesiologist supervising multiple cases to take over after positioning, stabilization, and completion of documentation [4]. Our generalizable finding is the calculation process (methodology) that other departments, with both anesthesiologists and nurse anesthetists present at induction and emergence, can use.

Our methodology would not be useful to departments that have sufficiently long turnover times that nurse anesthetists often can take breaks in between cases. Our methodology would apply to departments, such as the studied hospital, where cases are scheduled sequentially in rooms, with the rooms rarely ending far earlier than the end of the scheduled workday.

Finally, the methodology was developed originally to investigate organizational challenges in facilitating breast milk pumping sessions of nurse anesthetists and anesthesiologists [1,2]. As reported previously and shown in Table 1, many cases that have surgical times that are long enough in retrospect for breast milk pumping are not reliably of sufficient duration [1,2]. However motivated the person making the daily case assignments may be, there may be few good choices of operating rooms for nurse anesthetists or anesthesiologists returning from maternity leave to receive time for breast milk pumping sessions [1,2]. Especially based on our sensitivity analysis results for staff scheduling, we recommend departments take advantage of the multiple months of pregnancy and maternity leave to revise staff schedules so that lactating women can plan to work at facilities with long-duration cases and close to lactation rooms [27]. In addition, when staff scheduling is done, there needs to be a consideration for the additional practitioner(s) to give relief for breaks and breast milk pumping sessions.

## Conclusions

The feasibility of an anesthesiologist clinically supervising one, two, or three rooms to give lunch breaks to the nurse anesthetists in the rooms depends principally on how many rooms the anesthesiologist is supervising, the duration of the break, and the facility’s percentage of cases with surgical times longer than that duration. While specific numerical results will differ among departments, our results show that the precise time of a break should not be expected to be an important factor. Our methodology would be useful to other departments where anesthesiologists are clinically supervising independent practitioners, sometimes during cases long enough for a break, and when there is another anesthesiologist available for backup help. Such departments can use our methodology to plan staff scheduling for additional nurse anesthetists to give the remaining breaks.
